# *In vivo* and *in vitro* genome editing to explore GNE functions

**DOI:** 10.3389/fgeed.2022.930110

**Published:** 2022-09-27

**Authors:** Nili Ilouz, Avi Harazi, Miriam Guttman, Alon Daya, Shmuel Ruppo, Lena Yakovlev, Stella Mitrani-Rosenbaum

**Affiliations:** ^1^ Goldyne Savad Institute of Gene Therapy, Hadassah Medical Center, The Faculty of Medicine, The Hebrew University of Jerusalem, Jerusalem, Israel; ^2^ Faculty of Marine Sciences, Ruppin Academic Center, Michmoret, Israel; ^3^ Bioinformatics Unit of the I-CORE at the Hebrew University and Hadassah Medical Center, Jerusalem, Israel

**Keywords:** GNE, GNE myopathy, Gne KO muscle cells, Gne tagged mouse, cell cycle, dna damage repair pathway, CRISPR/Cas9, multi-omics analyses

## Abstract

GNE myopathy is an adult onset neuromuscular disorder characterized by slowly progressive distal and proximal muscle weakness, caused by missense recessive mutations in the *GNE* gene. Although the encoded bifunctional enzyme is well known as the limiting factor in the biosynthesis of sialic acid, no clear mechanisms have been recognized to account for the muscle atrophic pathology, and novel functions for GNE have been hypothesized. Two major issues impair studies on this protein. First, the expression of the GNE protein is minimal in human and mice muscles and there is no reliable antibody to follow up endogenous expression. Second, no reliable animal model is available for the disease and cellular models from GNE myopathy patients’ muscle cells (expressing the mutated protein) are less informative than expected. In order to broaden our knowledge on GNE functions in muscle, we have taken advantage of the CRISPR/Cas9 method for genome editing to first, add a tag to the endogenous Gne gene in mouse, allowing the determination of the spatiotemporal expression of the protein in the organism, using well established and reliable antibodies against the specific tag. In addition we have generated a Gne knock out murine muscle cell lineage to identify the events resulting from the total lack of the protein. A thorough multi-omics analysis of both cellular systems including transcriptomics, proteomics, phosphoproteomics and ubiquitination, unraveled novel pathways for Gne, in particular its involvement in cell cycle control and in the DNA damage/repair pathways. The elucidation of fundamental mechanisms of Gne in normal muscle may contribute to the identification of the disrupted functions in GNE myopathy, thus, to the definition of novel biomarkers and possible therapeutic targets for this disease.

## 1 Introduction

GNE Myopathy is an adult onset neuromuscular disorder, characterized by slowly progressive distal and proximal muscle weakness, and a typical muscle pathology (Argov and Mitrani-Rosenbaum, 2010). This disorder results from recessive mutations in the *GNE* gene. The *GNE* gene encodes the UDP-*N*-acetylglucosamine 2-epimerase/*N*-acetylmannosamine kinase (UDP-GlcNAc 2-epimerase/ManNAc kinase, GNE), a 753 aa protein highly conserved in mammals. GNE consists of two functional domains, an N-terminal epimerase domain and a C-terminal kinase domain, which are the key enzymes in the biosynthesis pathway of sialic acid (Hinderlich et al., 1997; Effertz et al., 1999). Several mutation types have been reported in GNE myopathy patients, the vast majority being compound heterozygote missense mutations dispersed along the entire gene, both in the epimerase and in the kinase coding sequences, bi-allelic at either domain or combined. Although some cases of heterozygous null and missense mutations have been described, interestingly, no double null mutations have been reported to date in any GNE Myopathy patient. This observation is in line with the hypothesis that GNE is essential for embryonal development and supported by the fact that GNE knockout is embryonically lethal in mice at day E8.5 (Schwarzkopf et al., 2002). GNE is ubiquitously expressed in most tissues, but particularly in muscle the expression is very low. Although one report showed that marked GNE reduction does not occur in GNE Myopathy patients (Krause et al., 2007), the lack of reliable antibodies to detect endogenous expression that is very low in tissues strongly impairs the understanding of the localization and function of wild type and mutated proteins in muscle. At present, the pathophysiological pathway leading from *GNE* mutations to the muscle phenotype in GNE Myopathy is still unclear. The obvious hypothesis of impaired sialylation in patients’ muscle cells is still in debate. Some patients have reduced sialylation (general or of specific proteins) and others have not (Hinderlich et al., 2004; Noguchi et al., 2004; Saito et al., 2004; Salama et al., 2005; Broccolini et al., 2010; Sela et al., 2020). The established mouse models are also difficult to interpret. Hyposialylation was detected in the M743T knock-in mouse model (the founder mutation in the Middle Eastern cluster), which presents a severe renal phenotype (Galeano et al., 2007; Sela et al., 2013; Benyamini et al., 2020) but no symptoms in muscle. Although treatment with sialic acid or its metabolic precursors, e.g., ManNAc, could rescue (at least in part) the renal failure in these mice, the lack of renal involvement in GNE myopathy patients and the lack of muscle phenotype in these mice, hamper conclusions on GNE myopathy mechanism and treatment. Finally, a transgenic mouse overexpressing the human GNE epimerase D207V mutation, one of the prevalent mutations among Japanese patients, was generated on a Gne endogenous knock out background (Malicdan et al., 2007). These Gne^(−/−)^ hGNED207V-Tg mice were reported to show hyposialylation in most organs, and also muscle phenotype, that could be partly improved by administration of ManNAc, the product of the epimerase activity in the sialic acid pathway (Malicdan et al., 2009). However, this model was not consistent and is controversial (Crowe et al., 2022; Mitrani-Rosenbaum et al., 2022). All these failed attempts for generating a GNE Myopathy animal model strongly emphasize that the process by which GNE mutations lead to myopathy is certainly not well understood.

Some studies by us and others have pointed to possible novel functions of GNE in the cell (Wang et al., 2006; Amsili et al., 2008; Singh and Arya, 2016; Harazi et al., 2017). In particular we have described impaired apoptotic signaling in patients cells (Amsili et al., 2007; Harazi et al., 2015) and identified the subtle involvement of mitochondrial processes in GNE Myopathy pathophysiology by microarray studies of patients’ biopsies (Eisenberg et al., 2008). In addition, proteomic analysis identified the differentially expressed proteins as related mainly to ubiquitination, stress response, mitochondrial processes, cytoskeleton and sarcomere organization (Sela et al., 2011). However, the changes detected in these studies, performed on muscle cell cultures from patients carrying missense mutations, were very mild. Since the available GNE Myopathy *in vivo* mouse models and *in vitro* cell culture models are less informative than expected, additional models where the effect of GNE mutation could be more dramatic are needed to determine the function(s) of GNE specifically in muscle. In the present studies we describe the generation of a mouse lineage where a FLAG tag has been fused to the endogeneous *Gne* gene to allow the spatio temporal follow up of Gne protein in muscle. In addition, we have used the Crispr/Cas9 technology to establish muscle cells lineages knocked out for the *Gne* gene, in order to elucidate possible novel function(s) of GNE in muscle.

## 2 Materials and methods

### 2.1 Mice maintenance and follow up

Mice were maintained at the facilities of the Authority for Biological and Biomedical Models at the Faculty of Medicine of the Hebrew University of Jerusalem. All animal procedures were performed in accordance with institutional guidelines under protocols approved by the Institutional Committee for Animal Care of the Hebrew University-Hadassah Medical Center. The mice were followed up for general behavior and weight. At various time points mice were euthanized and different tissues were collected and fixed in 4% paraformaldehyde. Muscles (gastrocnemius, quadriceps, tibialis anterior) were snap frozen in liquid nitrogen cooled in isopentane and stored at −80°C till processing for tissue sections (8 μ) and H&E staining.

### 2.2 CRISPR Editing of the Gne Locus

To generate the *Gne* ^Flag^ mouse two gRNAs targeting the carboxi-terminus of the Gne gene were designed, using CrisprGold and IDT gRNA designer softwares, which minimize off target outcomes. The well-established crRNA:tracrRNA Crispr/Cas complex strategy was followed (*idtdna.com*). Synthetic crRNA FLAG1 sequence TTC​TGG​ACT​ACA​CAA​CGC​GC and FLAG2 sequence CAC​AAC​GCG​CAG​GAT​CCA​CT were annealed with universal tracrRNA to generate the crRNA:tracrRNA complex. For HDR we designed a symmetrical, single-strand oligonucleotide donor containing the 3× FLAG sequence (ssODN; IDT Technologies) The ssODN was of the same strand of the gRNA to prevent hybridization of the two in the injected embryo. Approximately 200 CB6/F1 zygotes were microinjected with 0.61 μM guide RNA (cr + tracr complex), 0.3 μM Cas9 IDT protein and 15 ng/μl ssDNA FLAG donor. Viable 2-cell stage embryos were transferred to pseudopregnant CB6/F1 females. This procedure was performed at the Department of Veterinary Resources at the Weizmann Institute, Israel.

### 2.3 Gne^Flag^ Mouse genotyping

Four-week-old founder pups were ear punched and genomic DNA was extracted using a D-tail kit (Syntezza, United States). Each sample of extracted DNA (3 μl) was mixed with 9.5 μl nuclease-free-water, 12.5 μl Red Taq Mastermix (HighQu), and 1 μl each of primers 129SVF (5′ctg​gtg​atc​ctg​tct​gga​gtc​c 3′) and 129SVR (5′cac​tga​gct​gtc​tca​gca​gc 3′) flanking the C-terminus of Gne. PCR conditions were: 1 cycle at 95°C for 4 min., followed by 35 cycles at 95°C (30 s), 56°C (25 s), and 72°C (25 s), and a final 4-min. cycle at 72°C. Amplified products were sequenced and each positive founder mouse was backcrossed to assess germ line transmission.

### 2.4 Primary cell culture

Muscles samples of mice at various ages were cut into pieces and incubated in warm trypsin for 30 min at 37°C. After aggressive pipetting of the suspension, the supernatant was collected with the addition of 2% FCS, filtered through a 40 µ strainer, centrifuged and resuspended in DMEM supplemented with glutamine (2%), penicillin/streptomycin(1%) and 10% FCS. Cells were seeded on 10% matrigel precoated tissue culture plates for a week, then transferred to regular, non precoated culture plates. Medium was replaced every 1-2 days.

### 2.5 Gne^Flag^ protein immunoprecipitation and Western blot

Various tissues from the *Gne* ^Flag^ mouse and controls were harvested and homogenized using the TissueLyser LT (Qiagen) for 3 min at 50 Hz in RIPA lysis buffer (50 mM Tris buffer pH 7.5, 5 mM EDTA, 150 mM NaCl, TritonX100 1%, 1 mM DTT, 1 mM PMSF, 17 μg/ml Aprotinin, 10 μg/ml leupeptin, 1 mM Vanadate), followed by 20 strokes through a syringe with a 26 gauge needle, incubation on ice for 10 min., and centrifugation (14,000 rpm for 30 min at 4°C). Protein concentration was determined using Bradford Reagent (Sigma). For FLAG-IP, 150 μg of total protein Flag lysates were incubated for 4 h at 4°C with ready anti FLAG coated beads (Sigma); loaded beads were washed with PBS/0.3% Tween 20 and protein elution was recovered by boiling beads in 2X Laemeli sample buffer (Biorad). Protein extraction of Sol8 cells was done similarly. Protein lysates were separated on a precast 8% SDS-PAGE (Nusep, Australia) and transferred to 0.2 µm nitrocellulose membrane (PROTRAN BA 83; Schleicher & Schuell, Dassel, Germany) according to manufacturer instructions (Trans-Blot Electrophoretic Transfer Cell; Bio-Rad Laboratories, Hercules, CA, United States). Nitrocellulose blots were blocked in 5% milk for 1 h. *Gne* ^Flag^ was detected by incubation of the membrane in anti-FLAG M2 mouse monoclonal primary antibody (Sigma) diluted in 2% BSA/PBS-Tween 20 (0.3%) for 3 h at RT, followed by incubation in HRP-conjugated goat anti mouse secondary antibody (Jackson ImmunoResearch). Protein expression was visualized using the EZ-ECL (Biological Industries).

### 2.6 Sol8 cell cultures maintenance

Sol8 cells were purchased at ATCC. The diploid status of Sol8 cells, in particular for chromosome 4 where the Gne gene is located, was kindly assessed at the Department of Human Genetics, Hadassah Medical Center. Cells were maintained in DMEM, 2% Glutamine, 1% penicillin/streptomycin and 10% FCS or 2% horse serum for differentiation (Biological Industries, Israel).

### 2.7 Gne KO gRNAs design and injection

The gRNAs to knock out the *Gne* gene were designed according to the recommendations of the CrisprGold program (*crisprgold.mdc-berlin.de*). Three gRNAs targeting three different sites in exon 3 of Sol8 *Gne* were chosen and purchased as sense and antisense strands (Hylabs, Israel), at a concentration of 100 µM. For hybridization they were mixed together at a 1:1 ratio with 10x PCR buffer and incubated at 95^°^c for 5 min, at a final concentration of 10 µM. Each gRNA was ligated into the 2.c. PSpCas9 (BB)-2A-Puro (PX459) V2.0 plasmid (Addgene, kindly provided by Dr Reubinoff’s group, Hadassah Medical Center) at the BbsI restriction sites downstream of the U6 promoter. This plasmid carries also the Cas9 gene, and the puromycin resistance gene for selection of transfected Sol8 cells. The three PX459 plasmids containing the three different gRNAs were transfected onto Sol8 cells (seeded on six well plates, 2. 10^5^ cells per well). The cells were transfected either with two or with all three gRNAs using Lipofectamine^®^ 2000 DNA transfection reagent (Cat#11668027, Invitrogen, United States). After 24 h, puromycin (3 μg/ml) (Cat#58582, Sigma, United States) was added for 48 h, then cells were washed twice with PBS and growth medium was added to each well for cell recovery. After 48 h, about 10 surviving colonies were expanded for each sample.

Each expanded colony was collected by trypsin treatment, DNA was extracted from the pellets by the D-Tail Mouse Tail DNA Extraction Kit (Cat#310801, Cat#310802, Syntezza, Israel). DNA from each colony was screened for the relevant changes by PCR and sequencing.

### 2.8 RNA extraction

RNA was extracted from each clone by the Tri Reagent isolation solution (Cat# TR 118, Molecular Research Center, United States) both for transcriptomics and for cDNA synthesis. RNA was reverse transcribed using the M-MLV Reverse Transcriptase kit (Cat#M1701, Promega, WI) according to the manufacturer’s protocol, and screened for the relevant changes by PCR and sequencing.

### 2.9 Polymerase chain reaction and sequencing

PCR was performed using the Veriti 96 well Thermal Cycler (AB Applied Biosystems, Israel). The PCR products were sequenced at the National Center for Genomic Technologies, Givat Ram, Jerusalem. Sequences were analyzed and compared using the DNA Star Seqman software.

### 2.10 Confluence assay

The test was performed using IncuCyte^®^ S3 Live-Cell Analysis System (Cat# No. 4763, Sartorius, Israel), which measures the percentage of cells’ confluence over time. Cells were seeded at various densities and followed up for several days, as pointed out for each experiment. Cells could also be visualized at any time point during the entire follow up. By using the cell-by-cell mode of analysis, the average cell size was evaluated for each cell population, according to the IncuCyte^®^ S3 manufacturer guidelines (Guidelines for the IncuCyte^®^ Cell-by-Cell Analysis Software Module).

### 2.11 Immunostaining

Cells were seeded on glass coverslips slides and were grown as indicated for each experiment. Following two washes with PBS, cells were fixed with 4% paraformaldehyde (Cat#BN15710, Barnaor, Israel) for 15 min at RT and then washed with PBS. For staining of intracellular epitopes (F-actin, *a*-actinin2, FLAG, γH2AX, 53BP1, GNE), cells were incubated for 20 min at RT with blocking and permeabilization PBS solution, containing 3% BSA, 1% donkey serum (DS) and 0.2% TritonX100 (Cat#T9284, Sigma, Israel). Cells were then incubated for 1–2 h at RT with the relevant primary antibodies diluted in PBS supplemented with 1% BSA, 1% DS and 0.02% Tween 20 (Cat#9005-64-5, Sigma, Israel) or in CAS-block (Cat#ZY-008120, Rhenium, Israel): mouse anti-FLAG (1:500, #F1804, Sigma, Israel), rabbit anti-GNE (1:2000, #25079-1-AP, Proteintech), mouse anti-γH2AX (1:300, #05-636, Millipore Israel), rabbit anti-53BP1 (1:400, #IHC 00001, Bethyl Israel), rabbit anti-α-actinin 2 (1:400, #7H1L69, Invitrogen), Phalloidin-Rx (1:5,000, R415, ThermoFisher). After three washes with PBS-0.05% Tween 20, cells were incubated for 30 min at RT in the dark, with the relevant secondary antibodies: goat anti-mouse Alexa Fluor 488 (1:350, #A-11017, Molecular Probes, Israel), donkey anti-rabbit Alexa Fluor 647 (1:150, #711-606–156, Jackson, United States), donkey anti rabbit Alexa Fluor 488 (1:350, #711-546–152, Jackson, United States), all diluted in PBS with 1% BSA, 1%DS and 0.02% Tween 20. After washing with PBS-Tween-20, cells were incubated for 15 min at RT with 0.1μg/ml DAPI (Cat#28718-90–3, Sigma, Israel) diluted in PBS. After two additional PBS washings, stained cells were covered by mounting solution (Cat# 17,985-01, Barnaor) and covering glass, then stored at −80^O^C. Secondary-only stained samples were used as controls for nonspecific staining for each secondary antibody on each type of cells. The analysis was done with a Nikon’s A1R + confocal microscope (Cat#2CE-SCNH-2, Nikon, Japan) and image analysis software—Image-Pro Plus 7 (Media Cybernetics, United States).

### 2.12 Flow cytometry analyses of Sol8 cells

Cells were detached and dissociated to single-cell suspension, immunostained and analyzed by CytoFLEX Flow Cytometer, (Beckman Coulter, Israel), using CytExpert software.

#### 2.12.1 PSA expression

Sol8 cells WT and GneKO were grown for at least 3 days without serum, then detached from the culture dish using EDTA, washed with PBS, and incubated with anti PSA antibody (1:100; kindly provided by Dr Gerardy Schahn) in FACS buffer (1%BSA in PBS) for 1 h on ice, washed and incubated for 30 min in the dark with goat anti-mouse Alexa Fluor 488 (1:150, #A-11017, Molecular Probes, Israel) secondary antibody. After washing, cells were resuspended in FACS buffer.

#### 2.12.2 Intracellular markers (g-H2AX, Ki67, 7AAD)

WT Sol8 and GneKO cells were detached from the culture dish using trypsin, washed with PBS, counted and were then fixed and permeabilized using ice cold 70% ethanol for at least 2 h at −20°C. After PBS washing, the cells were stained for 30 min on ice with the relevant antibody/staining solution (1:50 Alexa Fluor 488 conjugated anti γ-H2AX #BLG-613406, BioLegend; 1:50 rabbit anti-Ki67 #275R-14 Cell Marque; 1:25 7AAD staining solution #00-6993-50 Invitrogen). Following washing, the Ki67 stained cells were incubated in the dark for 30 min on ice with donkey anti rabbit Alexa Fluor 488 (1:150, #711-546-152, Jackson, United States).

#### 2.12.3 Propidium Iodide (PI) staining

Cells were detached and dissociated to single-cell suspension and were resuspended in FACS buffer supplemented with PI (1∶500 of 1 mg/ml solution, Sigma).

*p* values for all relevant assays were calculated using the Student’s T-test.

### 2.13 AAV infection

Sol8 cells were grown in 12 well plates in DMEM containing 10% FCS for few days. Prior to infection with the AAVrh74.MCK.hGNE viral vector (Mitrani-Rosenbaum et al., 2022), cells were washed twice with PBS. The viral vector (1.10E6/ml) was added in 0.5 ml FCS free DMEM, incubated at 37°C for 3 h, followed by the addition of 0.5 ml DMEM supplemented with 10% FCS. The morphology and growth of the cells were monitored using the Incucyte system.

### 2.14 Transcriptomics

RNA was extracted from each sample, in triplicates. Extracted RNA sequencing was performed at the Genomic Applications Center at the Faculty of Medicine, Ein Kerem, Jerusalem.

Library preparation was done using KAPA stranded mRNA kit (Cat# KK8421, Roche, Israel). Libraries were quantified by Thermo Fisher Qubit and Agilent TapeStation, and sequenced by Illumina NextSeq 500, using the NextSeq 75 bp kit, Single End run. Bioinformatics was performed at the Bioinformatics Unit of the I-CORE at the Hebrew University and Hadassah Medical Center. Normalization and differential expression analysis were done with the DESeq2 package. Genes with a sum of counts less than 10 over all samples were filtered out, leaving roughly 20,500 genes (out of around 48,500). Quality control showed a high level of distinction between the conditions (probably due to the source of the samples being from cell lines). Therefore, significance threshold was taken as padj <0.05. Significant genes were further filtered to include only genes with a large enough effect. This was achieved by requiring a baseMean of at least five and an absolute log2FoldChange bigger than 7.5/sqrt (baseMean) + 0.9 (for highly expressed genes this means a requirement for the fold-change to at least two, while genes with a very low expression would need fold change about 10 to pass the filtering). These were the genes considered for further analyses. Data was deposited at the GEO repository (accession number GSE202046).

### 2.15 Proteomics

Triplicates of 20 millions cells per sample were grown and collected by trypsinization, stored at −80^o^ and sent as frozen pellets to the Technion Smoler Proteomic Center. The samples were digested by trypsin and analyzed by LC-MS/MS on Q exactive HFX (Thermo). The data was normalized and analyzed with MaxQuant 1.5.2.8 vs. the mouse Uniprot database and further with the Perseus software. The identifications are filtered for proteins identified in at least two samples in one of the groups with FDR <0.01. The heatmap of the proteins differential was done using the ANOVA test. The selected differential proteins were those with a *p* value < 0.05 and at least a 2 fold change. For phosphoproteomics, after trypsinization, samples were phosphoenriched on TiOx2 beads prior to LC-MS/MS analysis. For ubiquitination proteomics, after trypsinization, ubiquitinated peptides were enriched using KGG IgG prior to analysis by LC-MS/MS. Data was deposited at the ProteomeXchange via the PRIDE database (identifier PXD033985).

### 2.16 Enrichment analysis

For enrichment analyses, the differentially expressed genes and proteins were subjected to GeneAnalytics (Ben Ari Fuchs et al., 2016) and IPA software (Qiagen, Germany) programs.

## 3 Results

### 3.1 Generation of GneFLAG mice

**FIGURE 1:**
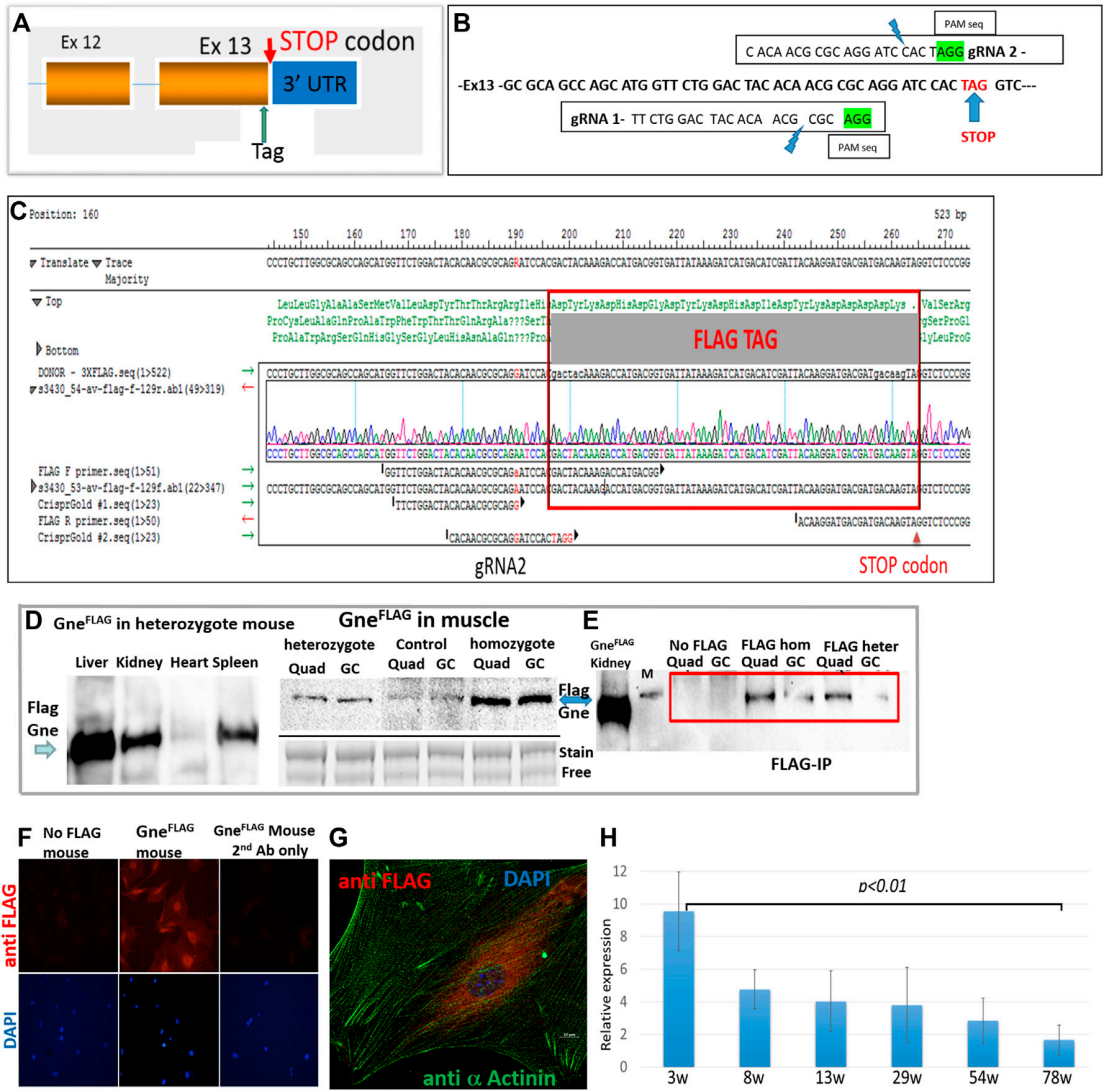
Generation of GneFLAG mice. **(A)**, schematic representation of the location of the incorporated tag; **(B)**, precise location of the various sequences relevant for Crispr/Cas9; **(C)**, sequence of the relevant region of a representative FLAG Gne generated mouse. **(D)**, Western blot of different tissues and muscles in GneFLAG heterozygote and homozygote mice. Control, mice with no FLAG, Quad, quadriceps; GC, gastrocnemius; stain free blot is shown for relative quantitation evaluation. **(E)**, Western blot after immunoprecipitation with anti FLAG Ab coated beads. M, 95kd band of the protein size marker. **(F)**, immunostaining of primary muscle cell cultures with anti FLAG Ab. **(G)**. Confocal section of a primary muscle cell immunostained with anti FLAG Ab. **(H)**, Relative quantitative representation of Western blots of the gastrocnemius muscle of mice sacrified at various ages (in weeks) with anti-FLAG Ab. Each time point represents the quantification of the corresponding western band relative to its respective actin value. The values are the average of 2 mice, each tested in triplicates.

Mouse *Gne* gene has several variants which differ in alternative splicing and initiation points, however they all end at the same last exon and same stop codon, as human *GNE* (Gene ID: 50798 and 10020 respectively). Therefore, tagging the carboxy-terminus of mouse endogenous *Gne* will detect all potential isoforms of the Gne protein, including the prevalent one which is 722 amino acids long (∼79 KDa). The cr:tracr CRISPR complex was used for adding the FLAG epitope tag as an homologous direct repair (HDR) mechanism at the last coding exon of *Gne*, just before the STOP codon (Figures 1A,B). Precise allele targeting and intact tag integration was verified by Sanger sequencing in 5/40 founders, with no additional alterations (Figure 1C). The *Gne* ^Flag^ allele was transmitted through the germline of the 5 founders at the expected Mendelian ratios. Homozygous *Gne* ^Flag^ mice were generated by crossing F1 siblings of different founders in order to increase the allele heterogeneity and minimize any unpredicted off-target homozygotization. Western blotting of heterozygotes and homozygotes *Gne* ^Flag^ mice with an anti-FLAG antibody revealed biallelic expression of the Gne protein (Figure 1D), since the signal detected in homozygote mice was stronger than in the heterozygote ones. Protein expression of Gne^Flag^shows a strong expression in liver, mild in kidney and spleen and faint in skeletal muscle and heart. The molecular weight of the FLAG tagged Gne was ∼82 KDa (744 amino acid) as predicted.

Since faint bands were detected in the muscles of mice with no FLAG (Figure 1D), most likely because overload of the blot (about 60 μg protein from muscle), in order to assess the specificity of the Gne^Flag^ protein detection we performed a pull down assay. Commercial Flag beads were incubated with protein muscle lysates of either heterozygous or homozygous *Gne* ^Flag^ mice. The immunoprecipitated proteins from each of the *Gne* ^Flag^ genotypes were blotted against FLAG antibody to validate genuine detection of the *Gne* ^Flag^ and rule out unspecific detection or antibody indirect interaction (Figure 1E). To note, all *Gne* ^Flag^ mice presented normal behavior and had a normal lifespan compared to their WT controls.

### 3.2 Primary cultures of GneFLAG muscle cells

Primary cultures were established from muscles extracted from the *Gne* ^Flag^ mice and were stained for GneFLAG expression. GneFLAG was detected in the cells (Figure 1F), in particular with perinuclear staining, as illustrated in Figure 1G and as previously described (Krause et al., 2007).

### 3.3 Spatio temporal expression of gne in muscle

To investigate Gne expression alongside the mouse lifespan, we harvested skeletal muscles at different time points, from early age—after weaning ended- and up to fully adulthood at the age of 18 months. In Figure 1H we can see that GneFLAG protein expression levels in gastrocnemius skeletal muscle is significantly highest at the youngest age and decreases as the mice matured. No significant differences were found in the mid-range ages, besides the edges.

This GneFLAG expressing mouse lineage will be useful as a platform for follow up of manipulated Gne expression *in vivo*, as there are several attempts to generate animal models for GNE Myopathy to identify novel biomarkers and for the assessment of potential therapies.

### 3.4 Generation of Sol8 muscle murine cells Gne KO

**FIGURE 2:**
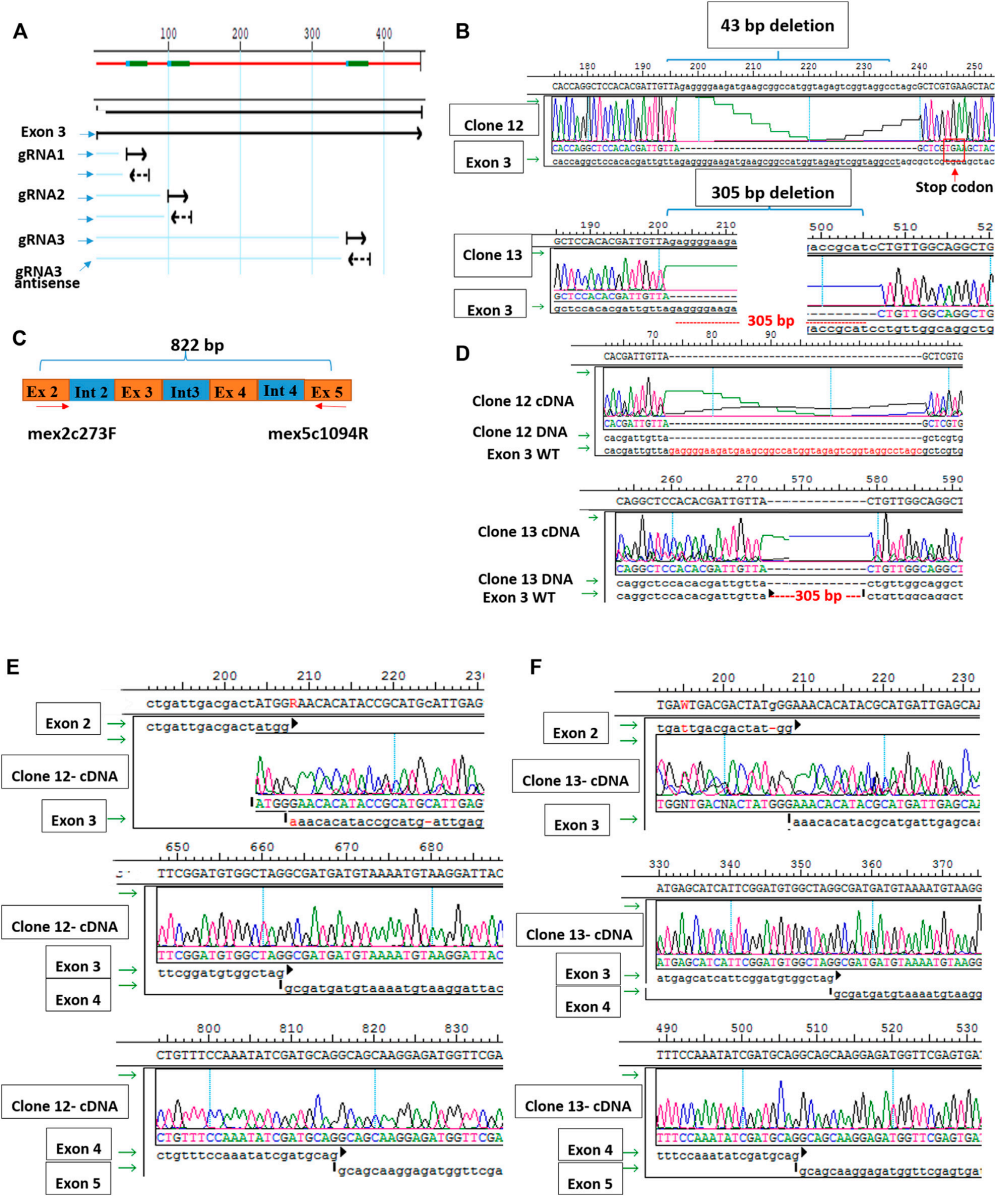
Generation of Sol8 Gne KO cells. **(A)**, schematic representation of the 3 gRNAs location in Gne exon 3. **(B)**, sequences of clone 12 and clone 13 DNAs, showing the respective deletions generated by Crisp/Cas9. **(C)**, schematic representation of the location of the primers used to test the cDNA sequences of clones 12 and 13. **(D)**, sequences of the cDNAs from clones 12 and 13 aligned with the respective DNA. **(E,F)**, sequences at the junction regions between the different exons in clone 12 and clone 13 respectively.

**FIGURE 3:**
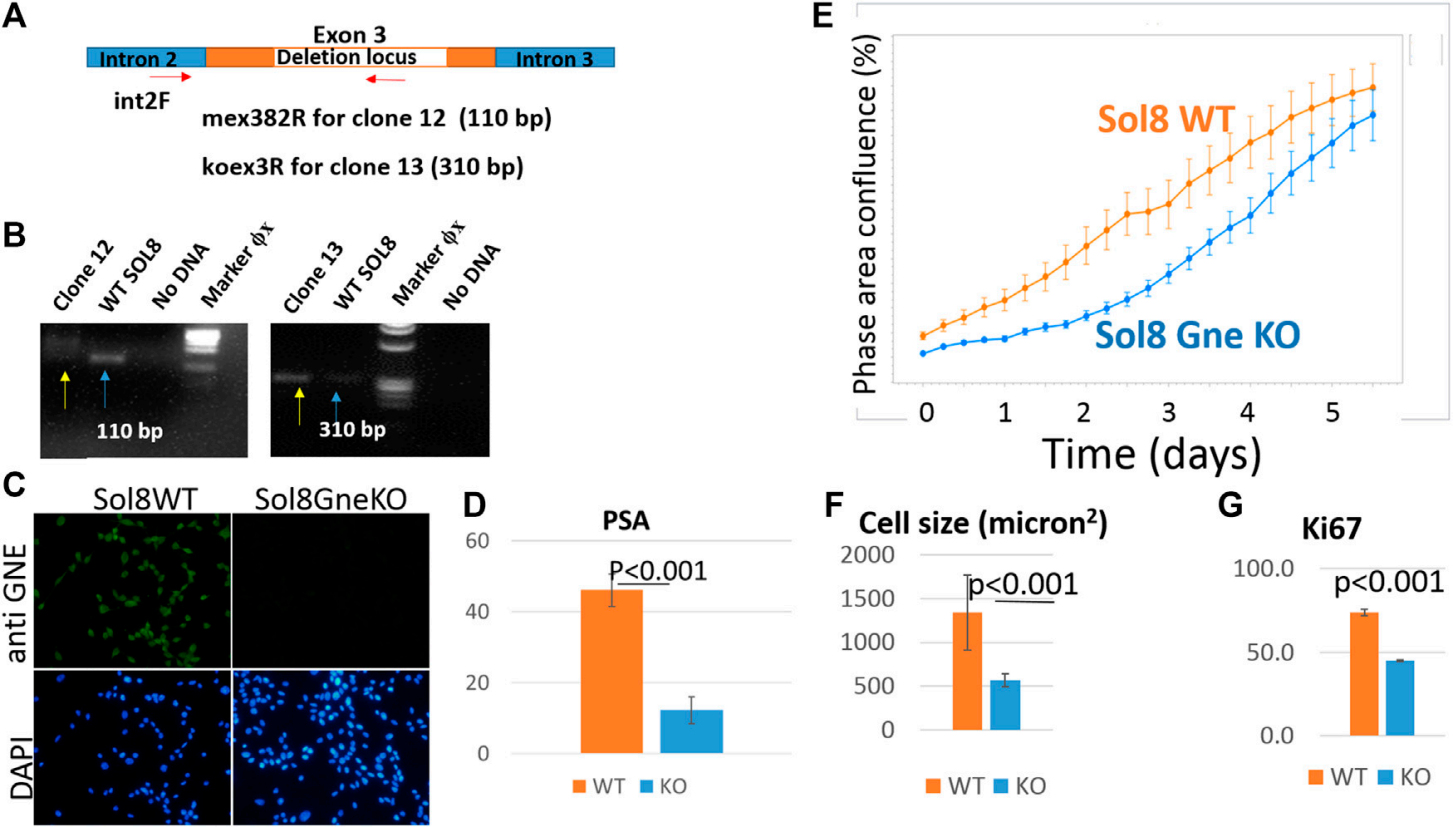
Validation of the GNE KO status in Sol8 manipulated cells. **(A)**, schematic representation of the location of the primers used to verify Gne KO homogeneity; **(B)**, agarose gels illustrating the PCR products of clones 12 and 13 (yellow arrows) compared to controls (blue arrows); **(C)**, immunostaining of Sol8 Gne KO cells with anti-GNE Ab. **(D)**, FACS analysis of PSA positive cells (% of positive cells) (*n* = 4 replicates); **(E)**, Incucyte confluency analysis; **(F)**, cell size determination (n > 300 cells); **(G)**, FACS analysis of anti Ki67 Ab immunostained Sol8 cells (% of positive cells) (n = 3 replicates).

Since muscle biopsies and cell cultures from GNE mutated patients are not very informative, and Gne KO is lethal in mice, we decided to establish Gne KO muscle cells. We generated Sol8 Gne KO murine cells by the Crispr/Cas9 method, using three guide RNAs designed to knock out exon 3, an early exon in *Gne* which is common to both isoforms, Gne1 and Gne2 (Figure 2A). Each gRNA was cloned in the PX459 vector and transfected onto the cells, either in combination with a second gRNA or all 3 together. Puromycin resistant isolated clones were expanded and subsequently screened for sequence alterations at the target site in their DNA and mRNA (cDNA). Two clones were retained, one with a 305 bp deletion and one with a 43 bp deletion, both resulting in several subsequent stop codons (Figure 2B). In order to validate the knock out of the GNE protein in the two clones we tested whether the same deleted sequence was present at the RNA level. PCR was performed on both cDNAs, using a forward primer on exon 2 (mex2c273F) and reverse primer (mex5c1093R) on exon 5 (Figure 2C). Sequencing of the PCR products showed that the sequence of the cDNAs are identical to their DNA matching sequence. First, the relevant deletion is present in the cDNA (Figure 2D), second, the junction areas between exon 2 and exon 3, between exon 3 and exon 4 and between exon 4 and exon 5 are intact (Figures 2E,F), thus lowering the possibility that the Crispr/Cas9 manipulation has altered the splicing properties in the mutated RNA. The homogeneity of the clones for Gne KO cells was validated by the absence of PCR products at the deletion loci (Figure 3A). As seen in Figure 3B, only clone 12, with the 43 bp deletion, was homogeneous, with no PCR product detected from the deleted locus. This clone was selected for further characterization. Immunostaining with anti-Gne Ab, that can detect Gne in cell cultures but not in tissues, most likely since expression in cell cultures is much higher, confirmed the cells did not express Gne (Figure 3C). In addition, we looked for a reduction in polysialic acid, which is typically bound to NCAM in muscle cells as a result of Gne enzymatic activity. FACS analysis showed a significant reduction of PSA in Sol8 Gne KO cells (Figure 3D).

### 3.5 Characterization of Gne KO Sol8 cells

Live cell imaging showed that Sol8 Gne KO cells reached confluency later compared to their Gne WT counterparts (Figure 3E). This result suggests that the Gne KO cells divide slower compared to the WT cells, although confluency does not necessarily reflects proliferation, but could also be related to the morphology and size of the cells. Cell-by-cell mode of analysis in the Incucyte system, 1 day after plating, showed that Sol8 Gne KO cells were significantly smaller than the WT cells (Figure 3F). In addition, staining with the proliferation marker Ki67 2 days after plating showed the Sol8 Gne KO cells were less proliferative (Figure 3G), suggesting that a bigger fraction of the Sol8 Gne KO cell population is in the G0 state of the cell cycle.

**FIGURE 4:**
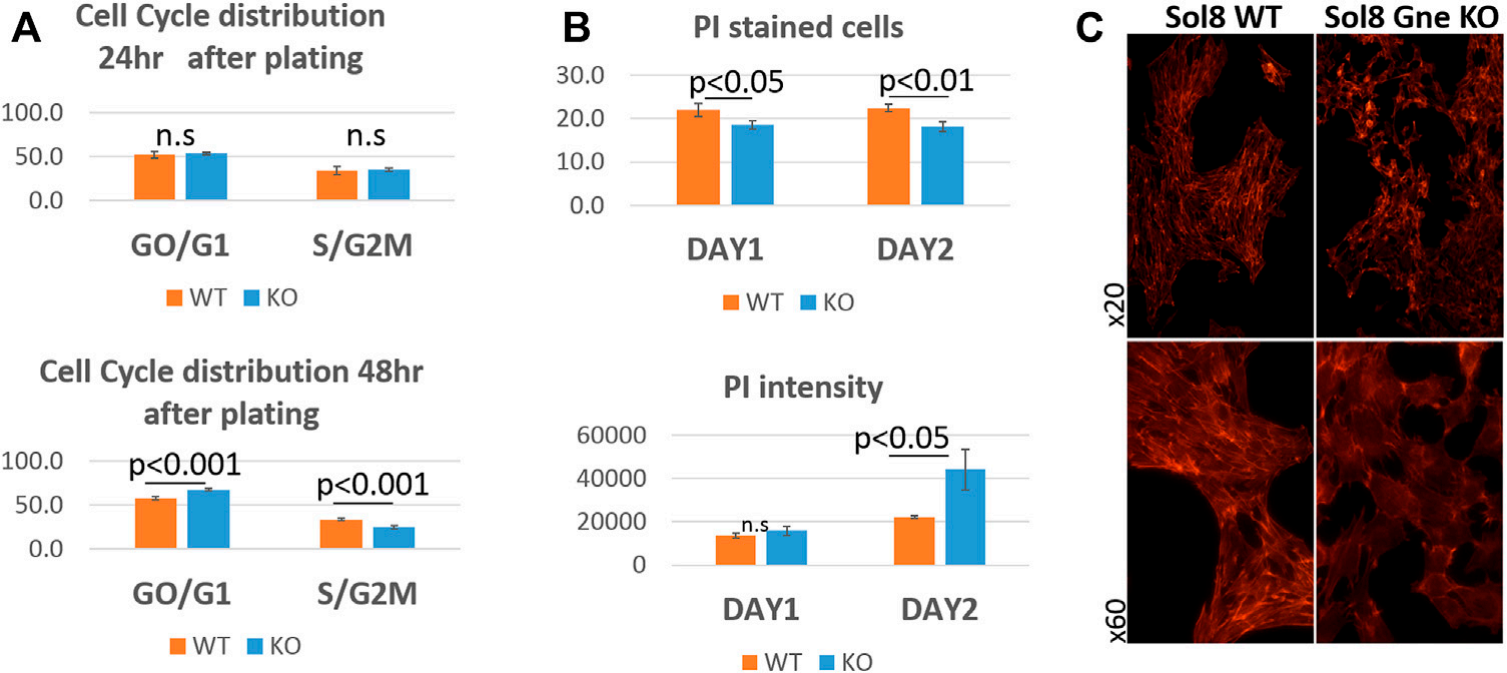
Cell cycle analysis of Sol8 Gne KO cells. **(A)**, determination of the cell cycle stages by FACS analysis of 7AAD stained cells at 24 h after plating (upper panel) and at 48 h after plating (lower panel) (% of positive cells) (*n* = 6 replicates). **(B)**, FACS analysis of PI stained (dead) cells: upper panel, % of positive cells; lower panel, staining intensity (MFI units) (*n* = 3 replicates). **(C)** Differentiated cells stained with phalloidin (F-actin).

To get more insights into the cell cycle process of the cells, we analyzed their DNA content. Flow cytometry using the 7AAD staining after permeabilization of cell and nuclear membranes, reflects the various stages of the cell cycle (Zelenin et al., 1984). No significant differences were observed 24 h after plating, however, after 48 h, more Sol8 Gne KO cells remained at the GO/G1 stage, and accordingly, less cells were at the S/G2M state, compared to the WT controls (Figure 4A). PI staining for dead cells showed lower percentage in the Sol8 Gne KO population (3%–4% lower) either after 1 or 2 days in culture. PI staining intensity is sensitive to the amount of DNA, therefore the higher intensity staining in the Gne KO cells on day 2 suggests the dead cells in the Gne KO population were at the S/G2M stage (Figure 4B). In order to test whether the defect in proliferation affects myoblast differentiation, myoblasts were cultured in differentiation medium. After 3 days, myotubes began forming in the Sol8 WT cultures, and long structures were seen 6 days post differentiation; however Sol8 Gne KO cultures were mostly single cells with no signs of fusion to each other and total absence of filament organization as seen by F-actin staining (Figure 4C).

**FIGURE 5:**
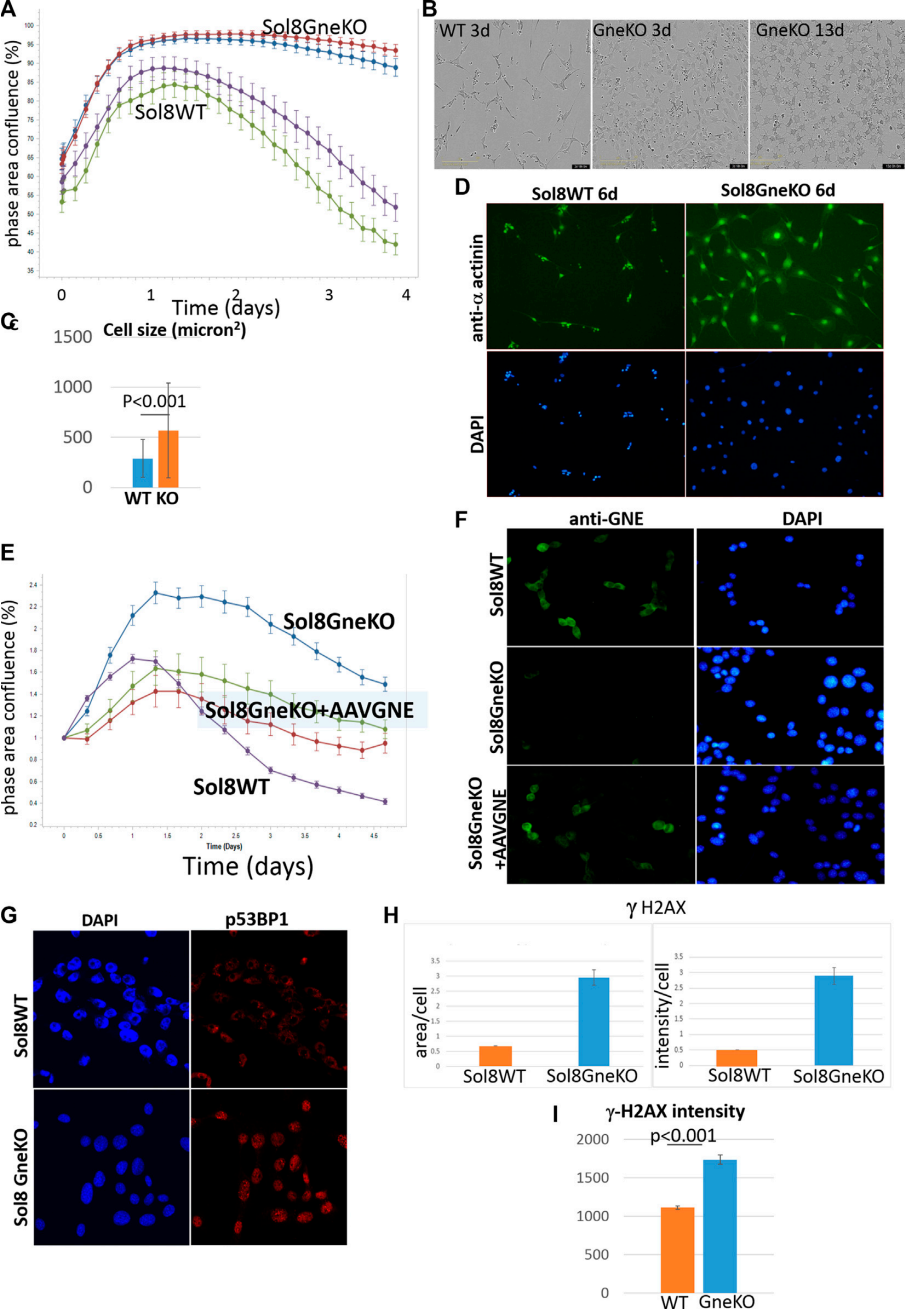
Sol8 Gne KO cells cultured in stress conditions. **(A)**, confluency curves determined by Incucyte (in duplicates; each point depicts the average of 9 fields). **(B)**. Morphology of the cells by phase contrast microscopy. **(C)**, determination of cell size at day 3 (*n* > 300 cells). **(D)**, staining of the cells with anti alpha actinin Ab. **(E)**, confluency curves of Sol8 Gne KO cells after AAVGNE infection. **(F)**, staining of the infected cells with anti GNE Ab. **(G)**, Immunostaining of Sol8 Gne KO and WT cells for p53BP1; **(H)**, Image -Pro Plus 7 analysis of area and intensity of γ-H2AX staining (relative units). **(I)**, FACS analysis of γ-H2AX stained cells (MFI units) (*n* = 3 replicates).

In an attempt to better evaluate differences between Sol8 WT and Gne KO cells, we cultured them in total absence of bovine fetal serum. Indeed, this condition pointed out distinct confluency features of the cells, as seen in Figure 5A: the Sol8 WT cells confluency graph rose for about 30 h, and then began to drop. Instead, for Sol8 Gne KO cells, the graph stayed stable for at least 4 days. The morphology of the cells was also different (Figure 5B): Sol8 WTcells were sparse and elongated (differentiated), while Sol8 Gne KO cells were more abundant, with a more rounded and flattened morphology already at day 2–3, and were much bigger (Figure 5C). At day 13, Sol8 Gne KO cells were alive, all flattened, while Sol8 WT cells did not survive (Figure 5B). Alpha actinin 2 and DAPI stainings illustrate the larger size of cells and nuclei in the Sol8 Gne KO cultures (Figure 5D).

#### 3.5.1 Phenotypic rescue by Gne supplementation

In order to assess that the differences between Sol8 WT and Gne KO cells were indeed the result of the absence of Gne, Sol8 Gne KO cells were infected by AAVrh74.MCK.GNE viral vectors. In these conditions, the phenotypic features of the infected cells were partially rescued and were closer to the Sol8 WT cells profile (Figure 5E). GNE presence in the infected cells was verified by immunostaining with anti-Gne Ab (Figure 5F). The partial and not complete rescue could be explained by the fact that not all cells were infected, or did not express GNE.

### 3.6 Transcriptome and proteome analysis

In order to broaden our knowledge on the potential functions of Gne in muscle cells, we analyzed the cells at the transcriptomic and proteomic level. These analyses were performed on proliferative (in the presence of 10%FCS) and on differentiated cells (2% horse serum), in order to compare between the proliferation state that simulates the satellite cells in the muscle tissue, and the differentiation state that should be physiologically closer to the more mature myofibers in the muscle tissue. The presence of sialic acid in the culture medium (in the serum) allowed to assign the differences detected more directly to the absence of Gne and less to the lack of sialic acid.

RNA and proteins were extracted from 3 independent cultures, and in triplicates for each sample.

#### 3.6.1 Transcriptomics

**FIGURE 6:**
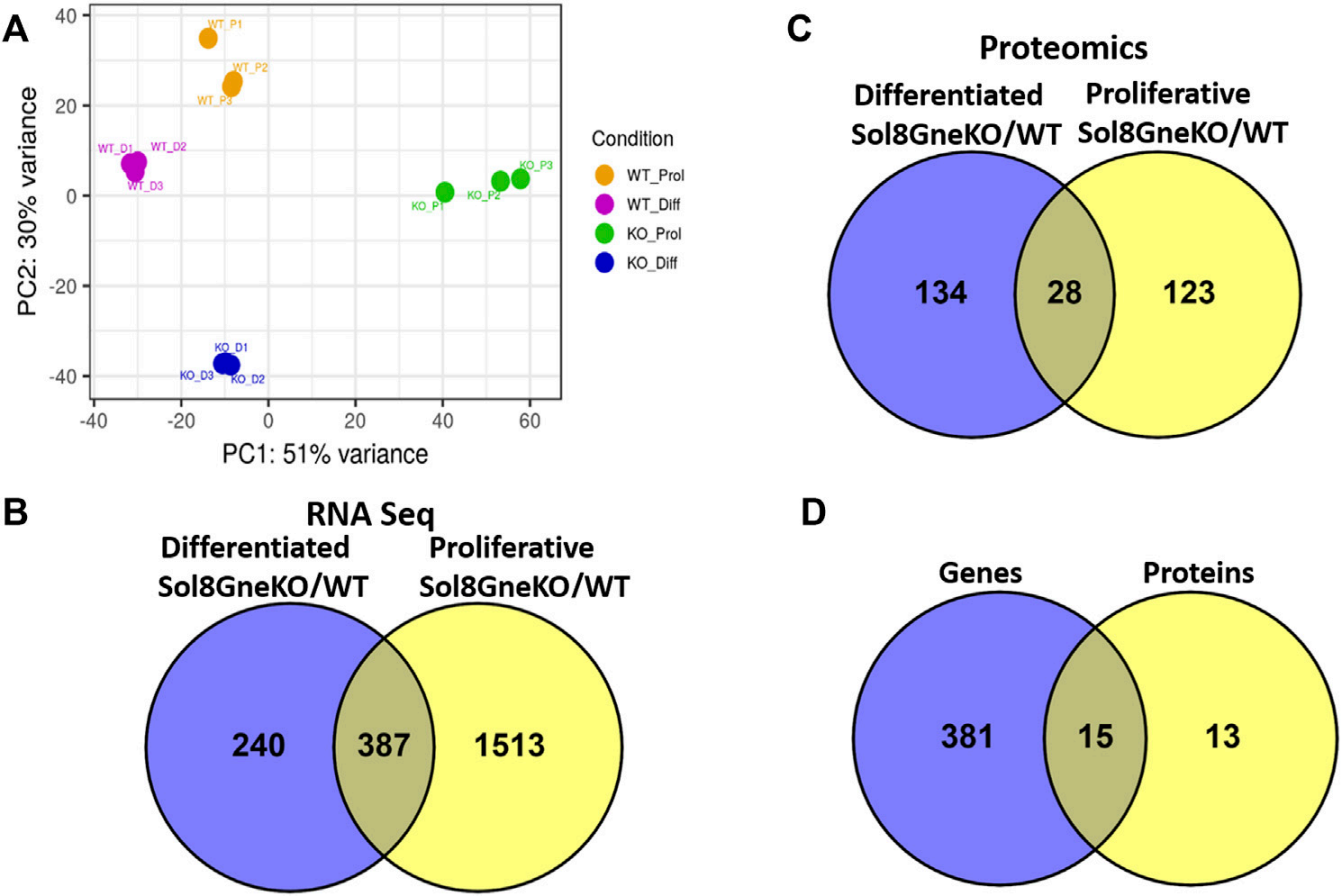
Genes and proteins differentially expressed in Sol8 differentiated and proliferative Gne KO versus WT cells. **(A)**, PCA plot for the RNA seq triplicate samples of: Sol8WT proliferative cells (WT_P1-WT_P3), Sol8 WT differentiated cells (WT_D1-WT_D3), Sol8 Gne KO proliferative cells (KO_P1-KO_P3), Sol8 Gne KO differentiated cells (KO_D1-KO_D3). **(B)**, Venny representation of Sol8 Gne KO differentiated and proliferative differentially expressed genes. **(C)**, Venny representation of Sol8 Gne KO differentiated and proliferative differentially expressed proteins. **(D)**, Venny representation of the differentially expressed genes and proteins in both differentiated and proliferative Sol8 Gne KO cell versus WT cells.

The expression profile generated by RNA sequencing shows that all cell categories are distinct from each other: the principal component analysis (PCA) display (Figure 6A) shows first, that the triplicates behave very similarly, and second, that there is a great variation between the WT and the Gne KO cells clusters, both for proliferative and differentiated cells. In addition, as expected, there is a significant difference between the proliferation and the differentiation expression profiles in both WT and Gne KO cells.

**FIGURE 7:**
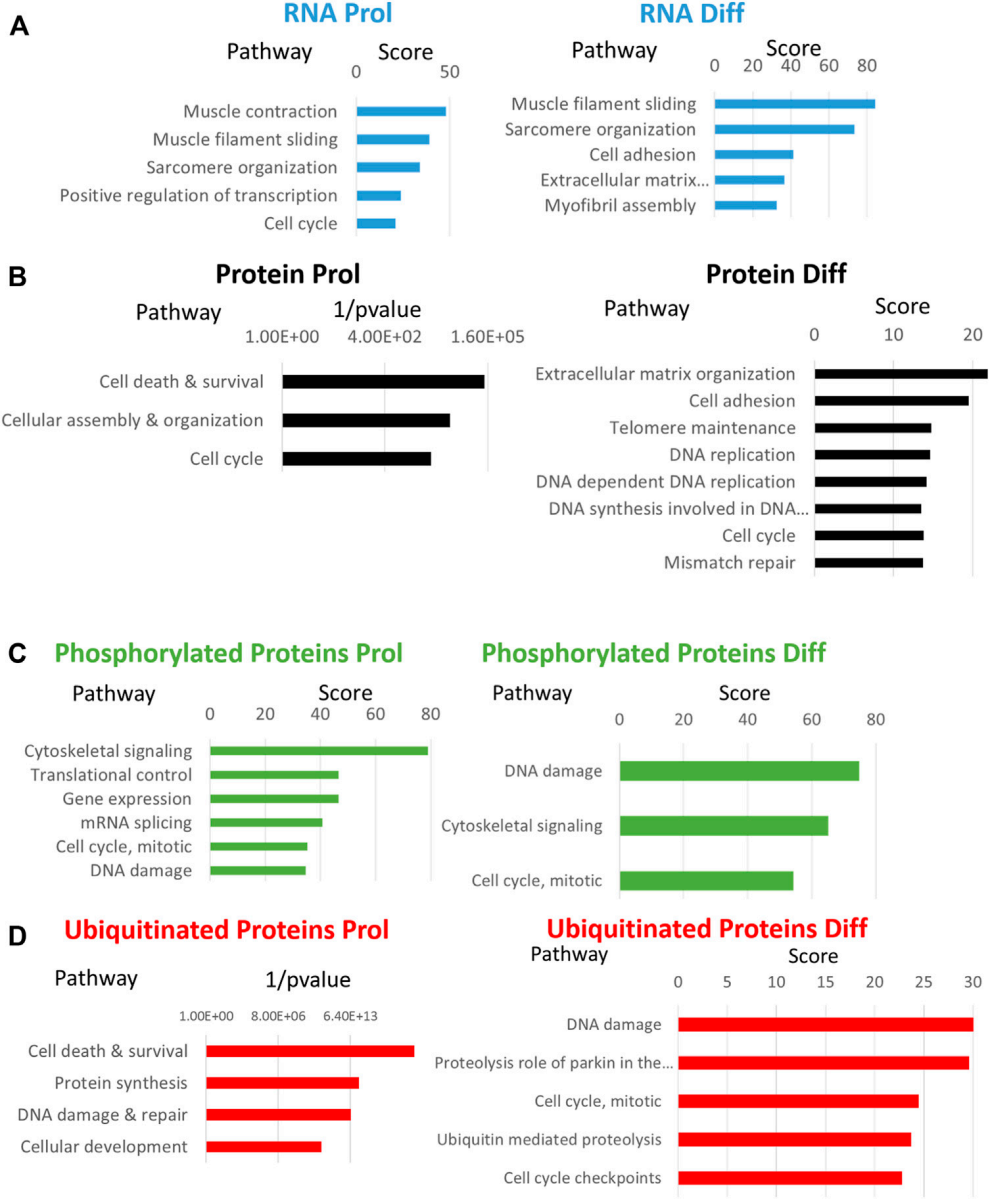
Enriched processes in Sol8 Gne KO versus Sol8 WT cells. Major enriched pathways in proliferative (Prol) and differentiated (Diff) Sol8 cells for RNA expression **(A)**, protein expression **(B)**, phosphorylated proteins **(C)** and ubiquitinated proteins **(D)**. Enrichment pathways represented were obtained with the GeneAnalytics software except for enrichment of proteins **(B)** and of ubiquitinated proteins **(D)** in the proliferative cells that were generated by the IPA software.

Comparative analyses of expressed genes between Sol8 Gne KO and WT cells were performed for both the proliferative state and the differentiated state. Significance threshold was taken as padj<0.05. Significant genes were further filtered to include only genes with a large enough effect, as explained in detail in the Methods section. Using these parameters 627 genes were identified to be differentially expressed (DEG) between Sol8 Gne KO and Sol8 WT differentiated cells; 1900 DEG between Sol8GneKO and Sol8WT proliferative cells (Figure 6B). Further analyses of the DEG showed enriched pathways in the Gne KO proliferative and differentiated cells, including sarcomere organization and cell cycle (Figures 7A,B). In molecular and biological terms, the affected functions included cellular assembly and organization, cell death and survival.

#### 3.6.2 Proteomics

Proteomics comparisons were performed between Sol8 GneKO and WT cells at both states, proliferation and differentiation (Figure 6C). To note, among the 4,857 proteins identified in the LC MS/MS process, the Gne protein was not detected in either the WT or the Gne KO cells, most likely because of the low expression levels of the protein. Enriched pathways among differentially expressed proteins in the proliferative and the differentiated state, pointed to the processes already found at the transcriptomic level, such as cellular and extracellular assembly and organization, and cell cycle (Figures 7C,D).

Interestingly, processes involving DNA damage and repair were also enriched in the Gne KO differential expressed proteins at the differentiated state. In an attempt to “capture” the primary effects of Gne lack in the cells, we looked at the common differentially expressed genes and proteins both in the proliferative and in the differentiated cells. A list of 15 genes came out of this analysis (Figure 6D), comprising mostly genes involved in cytoskeleton and myofibrillar organization -such as Fhl1, Gda, Jakmip2, Tagln, Postn, adrhgdib- and in cell cycle and DNA damage, such as Susd6, Tk1, Cd82, Cbr1 (Table 1).

**TABLE 1:** Common genes and proteins differentially expressed at both proliferative and differentiated states.

Gene Name	RNA Seq				Proteomics			
	^a^Diff.log2		^b^Prol.log2		^a^Diff.log2		^b^Prol.log2	
	FoldChange	padj	FoldChange	padj	FoldChange	*p* value	FoldChange	*p* value
Aldh3a1	0.954	0.000	2.409	0.000	1.660	0.002	4.101	0.001
Arhgdib	−3.517	0.000	−1.956	0.000	−5.801	0.037	−8.284	0.000
Cavin2	−2.659	0.000	−3.743	0.000	−3.686	0.000	−7.484	0.000
Cbr1	−3.102	0.000	−2.194	0.000	−6.406	0.000	−6.264	0.000
Cd82	−0.681	0.000	0.557	0.005	−1.799	0.000	−1.885	0.000
Cenpv	−1.297	0.000	−0.692	0.002	−1.242	0.007	−1.125	0.013
Col1a1	−0.383	0.027	−1.915	0.000	−5.100	0.000	−5.956	0.001
Fhl1	−5.339	0.000	−3.708	0.000	−8.177	0.004	−10.396	0.000
Gda	1.203	0.000	−0.952	0.000	−5.100	0.000	−8.798	0.000
Jakmip2	1.700	0.000	1.966	0.000	9.914	0.000	−5.885	0.000
Myl4	−4.869	0.000	−3.610	0.000	−6.543	0.000	−3.981	0.000
Postn	−1.008	0.000	−2.236	0.000	−5.273	0.000	−5.026	0.001
Susd6	−0.205	0.038	−0.201	0.021	−6.396	0.000	−5.956	0.000
Tagln	−1.965	0.000	−0.632	0.000	−1.313	0.000	−1.591	0.000
Tk1	−0.406	0.020	0.884	0.000	4.610	0.017	1.164	0.023

a Log2 fold change between differentiated Sol8 Gne K0 and WT cells.

b Log2 fold change between proliferative Sol8 Gne KO and WT cells.

A more comprehensive proteomics analyses followed to include phosphoproteomics and ubiquitination data. As a whole, 1,209 proteins were differentially phosphorylated in the Sol8 Gne KO differentiated cells-1,148 were down phosphorylated - and 713 in the proliferative Sol8 Gne KO cells. Differentially ubiquitinated proteins between the differentiated Sol8 cells included 588 proteins, and 277 were accounted for between the proliferative cells. Notably, enrichment analyses of the differentially phosphorylated and also ubiquitinated proteins in the Sol8 Gne KO cells pointed to the involvement of processes very similar to the ones enriched in the general proteomics and transcriptomics analyses, mostly cell cycle and DNA repair related processes (Figures 7E–H). Interestingly, the proteins involved in DNA damage/repair such as Tp53bp1, BRCA1, MDC1, RIF1, Xrcc1, and Xrcc5 were hyperphosphorylated in the Sol8Gne KO cells. Notably, Brcc3 was downregulated in Sol8 Gne KO cells and indeed hyperubiquitinated (Table 2).

**TABLE 2:** Differentially phosphorylated and ubiquitinated proteins involved in the DNA damage/repair pathway.

Phosphorylation			Proteomics			Ubiquitination	
Protein	*p*-value	^a^Foldchange (log2)	Protein	*p*-value	^a^Foldchange (log2)	*p*-value	^a^Foldchange (log2)
Tp53bp1	0.0382	5.4373	Brcc3	0.0477	-0.168	0.01	1.34
Brca1	0.0076	1.6941					
Mdc1	0.0001	5.9756	**Ubiquitination**				
Rif1	0.0000	5.3030	Protein	*p*-value	^a^Foldchange (log2)		
Xrcc1	0.0000	6.3098	H2afx	0.00	−1.22		
Xrcc5	0.0144	1.1170					
H2afy	0.0298	1.4906					

a Log2 fold change between Sol8 Gne KO and WT cells

### 3.7 Validation of the DNA damage/repair process

The DNA damage/repair process unravelled here by the multi-omics analyses would be a novel mechanism related to Gne. In response to DNA double strand breaks, the histone variant H2AX is phosphorylated by DNA damage sensor kinases generating γ-H2AX at the break site (Kinner et al., 2008). This phosphorylation event is critical for sustained recruitment of other proteins to repair the break. The p53BP1 protein is also a key DNA repair factor (Williams and Schumacher, 2016). To validate the results related to this pathway, we tested p53BP1 expression by immunofluorescence staining of Sol8 Gne KO and WT cells (Figure 5G), which showed a stronger signal in Sol8 Gne KO cells. Using image analysis software (Image-Pro Plus 7) for γ-H2AX expression, the area and intensity of the staining signal were quantified (Figure 5H). The results showed that the γ-H2AX signal (area and intensity) was indeed higher in the Sol8 GneKO cells compared to the WT cells. FACS analyses supported this trend (Figure 5I). To note, the H2AX protein was found to be less ubiquitinated in Sol8 Gne KO compared to WT cells (Table 2).

## 4 Discussion

GNE myopathy pathophysiology has not been elucidated yet, in spite of the knowledge accumulated on GNE protein function as a bifunctional key enzyme in the biosynthesis pathway of sialic acid. Among the obstacles to the understanding of the disease mechanism is the lack of a reliable antibody for GNE detection in tissues, and the lack of an animal model that recapitulates the disease symptoms. Only one study in 2005 examined tissue distribution of the Gne protein and showed that the highest expression of Gne was in the liver with moderate levels in kidney and spleen (Krause et al., 2005). These findings could not be corroborated later, in experiments using Gne mutated mice, because all available antibodies since, used for tissue expression, can only detect high levels of Gne, that certainly were not present in the different skeletal muscles. All additional information on Gne expression comes from RNA profiling. Data from the Mouse ENCODE transcriptome database (https://www.encodeproject.org/comparative/transcriptome/) and from studies performed on *Gne* expression at the RNA levels, showed the highest levels in liver and the lowest expression levels in heart and various skeletal muscles. Lately, genome editing technologies have been broadly applied to introduce different genes or sequences at precise sites in the genome of cells or living animals. Here we have taken advantage of the genome editing technology Crispr/Cas9 to incorporate a FLAG tag precisely before the stop codon of the *Gne* gene in mice. By this means we have shown that GneFLAG protein expression on various murine tissues shows a similar pattern in direct correlation with the reported RNA levels. In addition we have shown an age dependent expression of Gne protein, higher at younger age and declining thereafter, consistent with the known high need of sialic acid at the fetal stages of development in all organisms (Schwarzkopf et al., 2002). We believe our findings are genuine and not as a result of off target mechanisms, since at the molecular level one of the main parameters for gRNAs design and selection was the absence of potential non specific targets. Furthermore we have minimized any unpredicted allele impairment since the characterized mouse lineage was the result of four generations of consecutive breedings in between the different Gne^FLAG^ founder mice, which maximize haplotype diversification. The generation of Gne^FLAG^ mice as presented in this study allows a precise assessment of Gne protein expression in all tissues, thus it could be used as a platform for a thorough elucidation of Gne protein levels and functions in different mice models. Indeed the generation of Gne mutated mice and of conditional muscle Gne KO mice using the Gne^FLAG^ mouse as the initial target are in progress in our laboratory.

In parallel with these *in vivo* studies, we have generated a lineage of murine muscle Sol8 cells knocked out for the *Gne* gene, and characterized these Gne KO cells for various altered molecular processes and biological pathways. Understanding the effect of *Gne* gene mutations on molecular pathways in the muscle cell can help to understand the mechanism of the disease. To date, only very mild effects in molecular pathways were observed in muscle cells from patients. Therefore, we reasoned that in identical systems, differing only by the presence/absence of GNE, there is a better chance to see significant changes in processes related to the *GNE* gene. Further, *Gne* knock out cells might unravel the mechanism that leads from *GNE* mutation to the myopathy. We chose to use Sol8 cells -a mouse myogenic cell isolated from primary cultures of soleus muscle- and not C2C12 cells (immortalized mouse myoblast cell line), which is commonly used in studies on muscle cells (Tang et al., 2018; Hwang et al., 2019), because C2C12 are immortalized cells with many chromosomal aberrations, in particular they are tetraploid for chromosome 4, thus contain four alleles of the *Gne* gene, while Sol8 cells are diploid, and present normal karyotype. To achieve our goal, the KO of the *Gne* gene should occur in all alleles, which, when using the Crispr/Cas methodology, will be easier to perform in diploid cells. In order to perform the KO of the *Gne* gene we used the Crispr/Cas9 genome editing method to disrupt the target gene, shifting the reading frame, which can result into an early stop codon (Kuscu et al., 2017; Manghwar et al., 2019; Yang and Huang, 2019). Exon 3 was chosen as a cleavage target for Crispr/Cas9 system, based on previous studies showing that knocking out exon 3 of *Gne* is enough to knock out the whole gene (Schwarzkopf et al., 2002; Malicdan et al., 2007). To increase the efficiency of the process to get homozygote KO, three gRNAs were designed in order to target three different sites at exon 3, taking in consideration that any alteration within these sites and even deletion of complete fragments, will lead to premature stop codons in the Gne frame. This strategy proved to be very efficient since among the 40 clones examined all showed sequence changes, mostly with deletions of different sizes located at the exon 3 targeted sites.

Among those relevant clones, we found at least two clones with homozygote deletions causing a frame shift which resulted in the generation of three premature stop codons close downstream: one clone with a 43 bp deletion as a result of the cleavage at the target sites of gRNA1 and gRNA2 and the second clone with a 305 bp deletion as a result of the cleavage at the target sites of gRNA1 and gRNA3 (and probably also of gRNA2). In order to verify that the specific deletions did not cause a change in the splicing signals in the *Gne* gene, we sequenced the cDNA of these clones and assessed that first, the relevant deletion is present in the cDNA, and second, that the junction areas between exon 2 and exon 3, between exon 3 and exon four and between exon four and exon 5 are intact, thus lowering the possibility that the Crispr/Cas9 manipulation has altered the splicing properties in the mutated RNA.

Another issue to address when genome editing cell cultures, is the risk of having a mixed population of both WT and knocked out cells. Indeed the selection of our clones was done according to their puromycin resistance. This selection addresses the transfection efficiency of the PX459 vector solely, but does not select for cells where the DNA was efficiently cleaved or those having premature stop codons as a result of deletions. Therefore, we can expect to have isolated clones with a mixture of cells, carrying the desired change but also cells with either different changes (that would not cause a genetic KO in our case) or even WT cells. Although the PCR products and subsequent sequencing showed a quite “clean” sequence background, thus indicating that at least the majority of the cells analyzed carry the relevant DNA sequence, it does not rule out that a small proportion of the cells does not. This could be important when examining the different features of the Sol8 Gne KO cells versus the WT cells, since the “dramatic” effects we are looking for could be attenuated by the presence of non Gne KO Sol8 cells. To evaluate the extent of the Gne KO genotype in the Sol8 cells population, whether our Gne KO clones are contaminated with WT cells, the strategy we followed was not only to check the sequence between the expected deletion site to validate the deletion itself, but rather to try and detect the cells that do not carry the deletion. For this purpose, we performed a PCR assay using a primer forward on intron 2 with a reverse primer located inside the deletion site, for each clone. The absence of a PCR product will suggest that there are very few or no WT contaminating cells in the clone. Indeed the results showed that in the clone carrying the 305 bp deletion, there were also cells that did not have the deletion. However, in the 43 bp deletion clone no PCR product was obtained, indicating that this clone contains a vast majority of cells with the deletion and very few, if any, contaminating WT cells. This was confirmed by the lack of staining in Gne KO cells with a Gne antibody able to detect Gne in WTcell cultures. After this verification, we continued our studies with this clone only. To our knowledge, this is the first muscle cell lineage which is KO for the *Gne* gene. The next step of the study was to assess the basic properties of these cells and to look for various molecular processes that could have been impaired.

In contrast to previous studies performed on human myoblast’ cultures derived from GNE Myopathy patients carrying the M743T mutation, which reported no differences in their proliferation potential compared to control myoblasts (Amsili et al., 2007), our results showed that murine Gne KO cells had a defect in cell cycle. A possible reason for this discrepancy could be the fact that the previous study was done on human muscle cells and this present study is performed on mouse cells. However, a more likely reason could be the fact that the M743T missense mutation has a relatively moderate effect on the proliferation of the cells. In contrast, the effect of knocking out the whole protein can be more pronounced. Thus, this result justifies our strategy to use Gne KO cells for our studies.

As expected, transcriptome and proteomic analyses revealed many genes and proteins differentially expressed between the Gne KO and WT cells, at both the proliferative and the differentiated states. Enrichment softwares pointed mostly towards the involvement of these genes in sarcomere and cytoskeleton organization in both the proliferative and the differentiated states, in RNA as well as protein expression. First, it is worth to note that there was a strong overlap between the pathways revealed for RNA expression and those represented in proteins. This is not always obvious, because of several reasons: first, the technology for the detection of RNA and peptides is completely different; in addition the level of expression in RNA and proteins is also completely different, so that it is not obvious that the molecules detected in RNA will also be detected as peptides and then proteins. The fact that in both analyses, the same main pathways are enriched basically reinforces the reliability of the findings.

Our results show that Gne has a role in biological and molecular pathways involved in the organization of murine muscle tissue. Indeed adult muscle is the result of very well orchestrated and synchronized processes where the myoblasts differentiate to myofibers which eventually will get organized as functional muscle fibers (Kuang et al., 2007). These results are in agreement with previous proteomics studies on human muscle cells of GNE Myopathy patients (Sela et al., 2011), although in those studies the effect was much milder. The involvement of GNE in these processes could explain the fibers’ atrophy in the muscles of the affected patients, as also seen in zebrafish depleted from gne (Daya et al., 2014). More unexpectedly, we also found that in Sol8 Gne KO cells the differentially expressed genes and proteins were significantly enriched for cell cycle and DNA damage/repair mechanisms. These specific pathways were also emphasized among the 15 differentially expressed molecules common to RNA and proteins, both at the proliferative and the differentiated state. Further, phosphorylation and ubiquitination analyses of the proteome in Sol8 Gne KO compared to Sol8 WT cells also point to enrichment of the very same cell cycle and DNA damage/repair pathways in the cells lacking Gne. This finding could also be precisely validated by staining with γH2AX and p53bp1, both hallmarks of this pathway, showing an actual increase in DNA double strand breaks in Sol8 Gne KO cells. This is the first report for such finding, which implies a role for Gne in the regulation of the cell cycle through the DNA damage-repair pathway. This could explain the cell cycle arrest (the long G0/G1 state) and the morphology observed in the Sol8 Gne KO cells (Barr et al., 2017; Krenning et al., 2019). This novel function of Gne in the cell could also be part of the GNE Myopathy disease mechanism. Impairment of the DNA damage/repair pathway could affect the continuous regeneration of muscle tissue, progressively resulting in muscle weakness and eventually leading to muscle atrophy The possibility that the DNA damage/repair pathway had been unspecifically activated following Cas9 activity is very unlikely since the cells were tested (by transcriptomics, proteomics and by staining) after broad expansion of the generated Gne KO Sol8 lineage, thus most likely after the complete disappearance of the original PX459 transfected plasmid producing the Cas9 protein. In addition, we have seen that supplementation of Gne in the cells rescues, at least partially, their phenotype. It is important to note that these studies have been performed on cells cultured in the presence of fetal bovine serum (when analyzed at the proliferative state at least), that is in the presence of sialic acid. Therefore the described findings can be assigned more directly to the lack of Gne specifically, and not to the absence of sialic acid. The only well-established known function of GNE is its key role in the sialic acid biosynthesis pathway. We have specifically looked at the molecular expression profile of the genes involved in this pathway (data not shown). A set of 30 genes included the five enzymes directly involved in the intracellular sialic acid metabolism (GNE, NANS, NANP, CMAS, and CMAH), two transporters that translocate CMP-sialic acid into the Golgi (SLC35A1 and SLC35A3), 21 sialyltransferases active in incorporation of sialic acid on sugar chains, UAP1, the enzyme leading to the synthesis of UDP-GlcNac, a step before the epimerase activity of GNE, and NAGK, a kinase which can also use UDP-N acetyl mannosamine as a substrate, thus competing with the kinase activity of GNE in the cell. Those enzymes were detected only at the RNA levels, but not in our proteomic studies. The only gene that changed significantly was St8sia2 (with a log2 fold change of −2.5), a sialyltransferase which incorporates sialic acid from CMP-sialic acid to N-linked oligosaccharides and glycoproteins, and is probably also involved in the production of polysialic acid (PSA). It could be that St8sia2 is more sensitive to the level of substrate than the other sialyltransferases (since it has to synthesize long polymers of sialic acid) and can sense more efficiently even a slight penury in sialic acid substrate, subsequently reducing its own expression. Indeed we also see in our cells a strong decrease in PSA. Therefore, it seems that as a whole, the absence of Gne in cultured cells does not affect directly the level of expression of all other molecules involved in neither the sialic acid biosynthesis nor the sialylation of the various glycoconjugates.

Indeed the novel role of GNE in cell cycle and DNA damage/repair mechanisms could also contribute to the progressive atrophy of the muscle fibers. Although GNE Myopathy is caused by missense mutations, Gne KO muscle cells might reveal GNE specific functions in muscle that could contribute to the understanding of the pathophysiology of GNE Myopathy. All these findings demand further investigation in patients’ cells to determine their precise mechanism and their relevance to muscle function and to the muscle-restricted pathology of GNE Myopathy.

## 5 Summary

CRISPR/Cas9 is an essential genomic tool for precise editing, facilitating development of disease models and even genetic therapies. In these studies we have used this method to establish a Gne^FLAG^ mouse lineage and a mouse muscle cell lineage knocked out for the *Gne* gene. These models allowed a better evaluation of the expression of *Gne* and of its role in various biological and molecular processes in the muscle cell as well as to assess the involvement of Gne in sarcomere organization. Along with the observation of Gne protein decrease with age in mice, for the first time we describe a role of the *Gne* gene in the cell cycle and DNA damage/repair pathways. Further studies based on these results could dissect the mechanism of this function more precisely. In particular this feature should be evaluated in muscle cells of GNE Myopathy patients. Certainly the combination of genome editing techniques, that are becoming more standardized, and multi-omics analyses is a powerful tool to open new avenues for the understanding of the mechanisms of GNE and GNE Myopathy.

## Data Availability

The datasets presented in this study can be found in online repositories. The names of the repository/repositories and accession number(s) can be found below: https://www.ncbi.nlm.nih.gov/, GSE202046 (RNA-sequencing); ProteomeXchange Consortium *via* the PRIDE partner repository with the dataset identifier PXD03398 (Proteomic)
